# The impact of being homeless on the unsuccessful outcome of treatment of pulmonary TB in São Paulo State, Brazil

**DOI:** 10.1186/s12916-016-0584-8

**Published:** 2016-03-23

**Authors:** Otavio T. Ranzani, Carlos R. R. Carvalho, Eliseu A. Waldman, Laura C. Rodrigues

**Affiliations:** Pulmonary Division, Heart Institute (InCor), Medical School, University of São Paulo, Av. Dr. Arnaldo, 455, 2° andar, sala 2144, Post-code 01246903 São Paulo, Brazil; London School of Hygiene & Tropical Medicine (LSHTM), Keppel Street, Room G9a, Post-code WC1E 7HT London, UK; Department of Epidemiology, Faculty of Public Health, University of Sao Paulo, Av. Dr. Arnaldo, 715, Post-code 01246904 São Paulo, Brazil

**Keywords:** Tuberculosis, Homeless, Treatment outcome, TB

## Abstract

**Background:**

Tuberculosis (TB) is a major public health problem requiring complex treatment, the success of which depends on biological, social, and institutional factors. São Paulo State (SPS), in Brazil, has a high TB burden. Because of high socioeconomic heterogeneity and chaotic urbanisation, homelessness might play an important role in the TB burden in SPS. Our aim was to determine the association between homelessness and outcome of treatment of pulmonary TB (PTB) in SPS.

**Methods:**

A historical cohort from the routine SPS TB database for 2009-2013 was analysed. The study population was newly diagnosed adult patients with PTB. Homelessness was ascertained at notification or when treatment started. Our outcome was unsuccessful outcome of treatment. We used logistic regression to adjust for potential confounders and multiple imputation for missing data.

**Results:**

We analysed 61,817 patients; 1726 (2.8 %, 95%CI 2.7-2.9 %) were homeless. Homeless patients were concentrated in bigger cities, were more frequently middle-aged males, had black/brown skin colour, and had received less education (P < 0.001, for all). Alcohol and drug use was three times more frequent in homeless patients (43.2 % vs 14.4 %, 30.2 % vs. 9.4 %, P < 0.001, respectively). HIV testing was less common among the homeless, of whom 17.3 % were HIV positive compared with 8.5 % among the not homeless population (P < 0.001). Microbiologic confirmation was more frequent among the homeless (91.6 % vs. 84.8 %, P < 0.001). Unsuccessful outcome of treatment was 57.3 % among the homeless and 17.5 % among the not homeless (OR = 6.32, 95%CI 5.73-6.97, P < 0.001), mainly due to loss to follow-up (39 %) and death (10.5 %). After full-adjustment for potential confounders, homelessness remained strongly associated with lower treatment success (aOR = 4.96, 95 % CI 4.27-5.76, P < 0.001). HIV status interacted with homelessness: among HIV-infected patients, the aOR was 2.45 (95%CI 1.90-3.16, P_interaction_ < 0.001). The population attributable fraction for the joint effect of homelessness, alcohol and drug use was almost 20 %.

**Conclusions:**

Confirming our hypothesis, homelessness led to a marked reduction in the successful treatment of newly diagnosed pulmonary tuberculosis. Homelessness and associated conditions were important contributors to lack of treatment success in pulmonary tuberculosis in São Paulo. A multifaceted intervention must be implemented to target this vulnerable population.

**Electronic supplementary material:**

The online version of this article (doi:10.1186/s12916-016-0584-8) contains supplementary material, which is available to authorized users.

## Background

Tuberculosis (TB) is an ancient infectious disease that poses several challenges from both the individual and the societal perspective [1]. The 2015 World Health Organization (WHO) report estimated 9.6 million new TB cases and 1.5 million TB deaths worldwide [2]. Although improvements in the burden of TB have been observed, several barriers to TB control remain [1–3].

Pulmonary TB (PTB) is the most frequent TB presentation, representing 80-85 % of cases [1]. PTB treatment requires prolonged and complex management [4]. Although there has been research on shorter treatment courses and alternative schemes, their use in clinical practice is still in debate [5]. Several factors influence treatment adherence, from high incidence of adverse drug events, to direct/indirect associated costs and stigmatization [3, 6–8]. Regarding TB treatment outcomes, some risk factors have been associated with unsuccessful outcome of treatment, such as: age [9–12], male gender [9, 13], race [10], being an immigrant [10], illiteracy [14], malnutrition [12], HIV positivity [9, 12, 14–17], chronic comorbidities [12, 13, 18], and socioeconomic factors [12, 16]. Some factors such as diabetes mellitus and smoking have been associated more frequently with relapse, treatment failure, and death [12, 19–21], while drug use and alcohol use were associated with loss to follow-up and death [15, 19]. Delay in diagnosis and treatment are also fundamental issues [9, 18, 22].

TB has been labelled a disease of poverty and health inequalities [3, 23]. Homelessness is likely to be an extreme life condition, encompassing several vulnerabilities that markedly increase the risk of being infected, having latent TB infection (LTBI) and developing active disease. Indeed, the homeless population has 10 to 85 times higher incidence of LTBI and active TB compared to the general population [24–26]. Homeless patients are also the source of TB outbreaks in shelters [27]. PTB among the homeless is usually highly infectious, due to the high burden of *Mycobacterium tuberculosis* in their sputum, delayed diagnosis and overcrowding [28]. These facts highlight the importance of a dedicated and multidisciplinary approach to these patients. However, there is a lack of epidemiological studies focusing on TB treatment outcomes in homeless populations [29–31]; few studies have addressed the impact of homelessness on treatment outcomes, and the available data came from high-income countries [25, 26].

Brazil is among the 22 countries with the highest number of TB cases. In 2014, PTB incidence was estimated as 44/100,000 [2]. São Paulo State (SPS) has the highest number of TB cases in Brazil (~20 %), with an estimated incidence of PTB of 37.7/100,000 in 2013 [32]. Incidence varies among cities according to size, population density and socioeconomic indicators [32–36] with higher incidences observed in bigger, more crowded cities, such as Santos (population ~400,000, incidence of 72.7/100,000) and São Paulo city (population ~ 11,000,000, incidence of 46.6/100,000) in 2013 [32]. Poorer areas and vulnerable groups have higher incidences [33, 37], such as four poor districts (incidence 149.0/100,000) in São Paulo city [35]. Treatment success for newly diagnosed PTB cases is around 80 % in SPS, thus not achieving the WHO goal of 85 % treatment success [2, 32, 38]. Although it is the wealthiest state in Brazil, the high socioeconomic heterogeneity and chaotic urbanisation in SPS, may mean that homelessness plays an important role in the TB burden [39].

The aim of this study was to determine the association between homelessness and the unsuccessful outcome of treatment of newly diagnosed PTB patients in SPS from 2009 to 2013. Our hypothesis is that newly diagnosed PTB patients with vulnerable conditions are at higher risk of not achieving treatment success. This study is justified because evaluations of PTB treatment outcomes among the homeless are rare; and a rigorous quantitative evaluation of this topic is missing for emerging countries.

## Methods

### Study site

The population of Brazil is 200 million, 22 % of whom (41 million) live in SPS [39]. The state has 645 municipalities with distinct characteristics. The Human Development Index (HDI) ranges from 0.639 to 0.862, and within the main city (São Paulo), the HDI ranges from 0.245 to 0.811. In 2003, a study reported that 27 % of the population of SPS lived in poverty, with marked income inequality (Gini index = 0.45) [39]. In Brazil, TB treatment is fully covered by the public health system. In SPS, following the National TB Program, directly observed therapy (DOT) is recommended for all patients. However, the final decision is shared between patients and multidisciplinary health staff [38]. DOT can bring additional support during treatment, such as food and transport vouchers [38, 40]. Although the national guidelines strongly recommend DOT for vulnerable groups such as homeless patients, there is no specific campaign to support its use among the homeless.

### Study design

A historical cohort from the routine electronic SPS TB database for 2009-2013 was studied.

### Study population

Newly diagnosed adult patients with PTB only. We included patients aged ≥15 years, who had never been treated for TB or who had taken anti-TB drugs ≤1 month (i.e. new TB cases) [2]. Before 2010, the Brazilian-TB Program considered patients treated more than five years earlier as new cases [38]. For the purpose of this study, only the first TB treatment was considered [2]. The “anatomical definition” of PTB from WHO includes patients with PTB associated with extra-pulmonary TB (EPTB) or miliary TB [41–43]. In order to have a homogenous population of new cases, we selected patients with PTB only.

We excluded presumptive TB patients whose diagnosis had changed during the follow-up period (i.e. they were not TB cases). We also excluded cases diagnosed and notified after necropsy (i.e. they would not have received treatment). Although the WHO “cohort definition” [41] includes patients with TB who died for any reason before or after starting treatment, we excluded patients diagnosed at necropsy because the study aim is to evaluate treatment success [11]. Finally, we excluded patients still on treatment at the moment of database acquisition.

### Exposure

A patient was considered homeless if they were without a fixed, regular, and adequate night-time residence at PTB notification or when treatment started. This definition includes individuals who live in emergency shelters/direct access hostels and those who live in places not meant for human habitation.

### Outcome

We used the 2013 WHO definitions to guide our main treatment outcome definitions [41], and adapted them to the SPS dataset. This classification consists of six outcomes, grouped into treatment success (cured or treatment completed) and unsuccessful outcome of treatment (treatment failure, death, loss to follow-up and not evaluated). We chose “unsuccessful outcome of treatment” as our primary outcome because we planned a pragmatic evaluation of a routine database.

### Confounding factors and interactions

Based on the literature, and on a theoretical framework, we selected potential confounders a priori to be adjusted for in order to obtain adjusted estimates of the impact of homelessness on PTB treatment outcome. Confounding factors included: age, gender, country of birth, race, education level, alcohol and drug use, diabetes mellitus, mental disorder, immunosuppression other than HIV, place of diagnosis, chest x-ray and microbiologic status at diagnosis, initial drug scheme and DOT.

For biological reasons, HIV is a major determinant of TB treatment outcome and we pre-specified that HIV is a strong effect modifier. We used the WHO 2013 definition [41], classifying HIV as positive, negative or unknown status (when the patient’s HIV status had been determined after notification, HIV status is routinely reclassified in the database).

### Data sources

The SPS-TB Program has had a specific strategy to improve its surveillance system since 1993. In this project we used the dedicated electronic system “TBweb” [44]. Since its inception, the SPS-TB Program has invested in the quality of data entry and maintenance of the consistency and validity of data. For instance, there is continuous audit and feedback, promotion of campaigns and rewarding of units for good data quality. We included data from 1 January 2009 to 31 December 2013 (dates of notification). The data were extracted on 31 October 2014.

### Plan of analysis

#### Sample size

SPS has 18,000-20,000 TB cases per year, totalling around 90,000-100,000 cases in our cohort [32]. Based on the TB Program reports, the estimated prevalence of homelessness among cases of tuberculosis is 2.5 % [32]. With 90 % power, type I error 5 % and 20 % unsuccessful outcome of treatment among those not homeless, our required sample size to evaluate the effect of homelessness was 3835 patients assuming 35 % or more poor treatment outcomes among the homeless. Sample size was calculated in Epi Info 7 (CDC-USA), using Fleiss with continuity correction [45].

#### Descriptive analysis

We described the patients’ general characteristics, showing the prevalence distribution of each variable. Categorical variables are shown as percentages and compared using the Fisher’s exact test or a χ^2^ test. For the exposure and primary outcome, we calculated their point-estimate measure and its 95 % confidence interval (CI) using the exact method.

#### Univariate and adjusted analysis

We used logistic regression models to evaluate the effect of homelessness on treatment outcome. Our main analysis was based on complete case analysis and we ran a sensitivity analysis using multiple imputation to deal with missing data.

We fitted univariate logistic regression models for the exposure and each potential confounding factor. To obtain an adjusted estimate of the association between homelessness and PTB treatment, we fitted a multivariate logistic regression. To build the final model, we included all a priori selected potential confounders. We also ran an additional final model to explore the interaction between homelessness and HIV status.

To conduct the multiple imputation, we first investigated the patterns of missing variables. We assumed the missing values to be missing at random (MAR) and explored whether they were conditioned on observed variables, suggesting a MAR mechanism [46].

We used a multivariate normal model and imputed the variables age, country of birth, race, education, place of diagnosis, chest x-ray, microbiologic status and DOT. We followed the recommended steps to build the imputed model [46], including all variables used in the final model as regular variables and the outcome. We also used auxiliary variables (year of notification, administrative region) and passive terms (HIV interaction). We generated five imputed datasets and, after the imputing process, we converted the continuous variables to categorical variables by “adaptive rounding” [47]. We combined the results using Rubin’s rule [48] and checked the convergence of the simulations by analysing the worst linear function (WLF) over successive iterations.

For all logistic regression models, we tested the hypothesis using likelihood ratio tests. For 95 % CI, we used the quadratic approximation of the log likelihood (Wald intervals). For the logistic regression models fitted in the multiple imputed data, we used Wald tests. In the fully-adjusted models, multicollinearity was assessed by the amount of variation on the standard errors of parameters on the logarithmic scale, comparing the model with and without the variable of interest.

We estimated the population attributable fraction (PAF) of vulnerable conditions from the final multivariate model by using the standard formula:

$$ PAF=p\hbox{'}\frac{\theta -1}{\theta } $$

where *p’* was the proportion of cases exposed and *θ* was the OR from the multivariable model.

All analyses were conducted in STATA 13.1 (StataCorp-Texas).

Further definitions and additional methodology are given in the online supplementary material (Additional file 1: eTable 1) [2, 41].

## Results

**Fig. 1:**
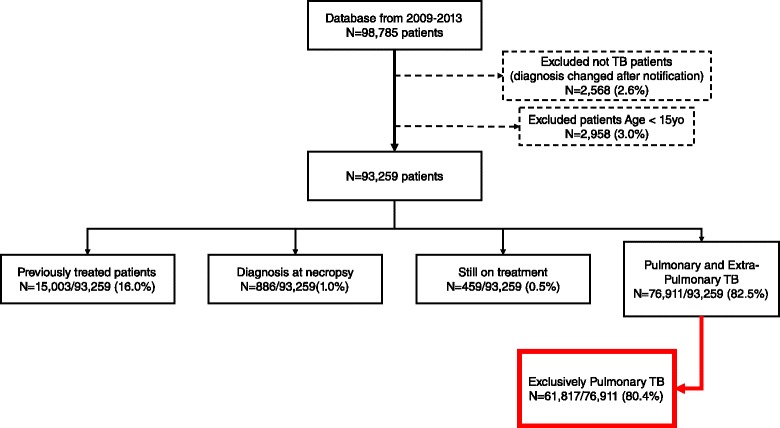
Study flow-chart

The flowchart is shown in Fig. 1. Of 93,259 adult TB patients, we excluded 16 % (15,003) because of previous treatment, 1 % (886) because they were diagnosed at necropsy and 0.5 % (459) because they were still on treatment. From the remaining 76,911 patients, we excluded 19.6 % (15,094) because they had EPTB. Therefore, we analysed 61,817 newly diagnosed patients with PTB only.

### General characteristics of the homeless

The prevalence of homelessness among PTB patients was 2.8 % (95 % CI 2.7-2.9 %; 1726/61,817). The majority of cases who were homeless were observed in big cities: four cities with more than 750,000 inhabitants had 5.5 % (1305/23,558) prevalence of homelessness and comprised 76 % (1305/1726) of all cases who were homeless.

**Fig. 2:**
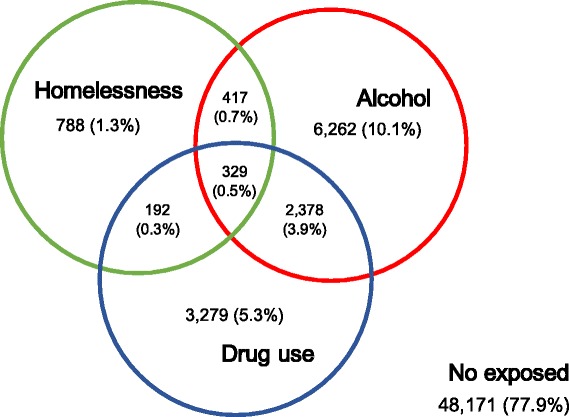
Intersection between homelessness, alcohol use and drug use

General characteristics are reported in Table 1. Homeless patients were more frequently middle-aged males (ratio male/female of 6.75), black/brown skin colour, and less educated (P < 0.001, for all). The prevalence of alcohol and drug use was three times more frequent in homeless patients (43.2 % vs. 14.4 %, 30.2 % vs. 9.4 %, P < 0.001, respectively). There was a marked intersection between homelessness and alcohol and/or drug use (Fig. 2). Diabetes mellitus was present in 6.3 % of patients, while mental disorders and “other immunosuppression” had a very low prevalence (1.9 % and 0.7 %, respectively). The prevalence of all such comorbidities was lower in the homeless patients, except for mental disorders. Overall, HIV testing was high (~85 %), but homeless patients were less frequently tested. The prevalence of HIV positivity was twice as high among homeless cases (17.3 % vs. 8.5 %, P < 0.001).

**Table 1:** Comparison of general characteristics of newly diagnosed pulmonary TB patients who were homeless and those who were not

	Values	Overall	Not Homelessness	Homelessness	P value
		(n = 61817)	(n = 60091)	(n = 1726)	
Age, years	15-25	12734 (20.6 %)	12616 (21.0 %)	118 (6.9 %)	<0.001
	25.1-35	16733 (27.1 %)	16301 (27.2 %)	432 (25.1 %)	
	35.1-45	11951 (19.4 %)	11417 (19.0 %)	534 (31.0 %)	
	45.1-55	10215 (16.5 %)	9794 (16.3 %)	421 (24.5 %)	
	55.1-65	6010 (9.7 %)	5833 (9.7 %)	177 (10.3 %)	
	65.1-75	2719 (4.4 %)	2683 (4.5 %)	36 (2.1 %)	
	75.1-85	1170 (1.9 %)	1168 (2.0 %)	2 (0.1 %)	
	85.1-105	230 (0.4 %)	229 (0.4 %)	1 (0.1 %)	
	Missing	55 (0.1 %)	50 (0.1 %)	5 (0.3 %)	
Gender	Female	17245 (27.9 %)	17023 (28.3 %)	222 (12.9 %)	<0.001
	Male	44572 (72.1 %)	43068 (71.7 %)	1504 (87.1 %)	
Country of birth	Brazil	50410 (97.2 %)	49151 (97.2 %)	1259 (98.7 %)	0.002
	Not-Brazil	1423 (2.8 %)	1406 (2.8 %)	17 (1.3 %)	
	Missing	9984 (16.2 %)	9534 (15.9 %)	450 (26.1 %)	
Race	White	26931 (51.6 %)	26396 (51.9 %)	535 (38.4 %)	<0.001
	Black	6106 (11.7 %)	5819 (11.5 %)	287 (20.6 %)	
	Mixed/Brown	18180 (34.8 %)	17615 (34.7 %)	565 (40.5 %)	
	Asian	556 (1.1 %)	549 (1.1 %)	7 (0.5 %)	
	Indigenous	444 (0.9 %)	443 (0.9 %)	1 (0.1 %)	
	Missing	9600 (15.5 %)	9269 (15.4 %)	331 (19.2 %)	
Education	Illiterate	1955 (3.9 %)	1886 (3.9 %)	69 (5.9 %)	<0.001
	1-3 years	6122 (12.3 %)	5920 (12.2 %)	202 (17.3 %)	
	4-7 years	19178 (38.5 %)	18651 (38.4 %)	527 (45.0 %)	
	8-11 years	18155 (36.5 %)	17821 (36.7 %)	334 (28.5 %)	
	12-14 years	3034 (6.1 %)	3005 (6.2 %)	29 (2.5 %)	
	> = 15 years	1327 (2.7 %)	1317 (2.7 %)	10 (0.9 %)	
	Missing	12046 (19.5 %)	11491 (19.1 %)	555 (32.2 %)	
Alcohol	No	52430 (84.8 %)	51450 (85.6 %)	980 (56.8 %)	<0.001
	Yes	9387 (15.2 %)	8641 (14.4 %)	746 (43.2 %)	
Diabetes mellitus	No	57955 (93.7 %)	56279 (93.6 %)	1676 (97.1 %)	<0.001
	Yes	3862 (6.3 %)	3812 (6.4 %)	50 (2.9 %)	
Drug users	No	55639 (90.0 %)	54434 (90.6 %)	1205 (69.8 %)	<0.001
	Yes	6178 (10.0 %)	5657 (9.4 %)	521 (30.2 %)	
Mental disorder	No	60673 (98.1 %)	58999 (98.2 %)	1674 (97.0 %)	<0.001
	Yes	1144 (1.9 %)	1092 (1.8 %)	52 (3.0 %)	
Other immunosuppression	No	61379 (99.3 %)	59656 (99.3 %)	1723 (99.8 %)	0.007
	Yes	438 (0.7 %)	435 (0.7 %)	3 (0.2 %)	
HIV status	Negative	47389 (76.7 %)	46399 (77.2 %)	990 (57.3 %)	<0.001
	Positive	5391 (8.7 %)	5093 (8.5 %)	298 (17.3 %)	
	Unknown	9037 (14.6 %)	8599 (14.3 %)	438 (25.4 %)	
Place of diagnosis	PHC/Ambulatory	40110 (65.9 %)	39199 (66.3 %)	911 (53.2 %)	<0.001
	Emergency service	13255 (21.8 %)	12661 (21.4 %)	594 (34.7 %)	
	Hospital	7499 (12.3 %)	7293 (12.3 %)	206 (12.1 %)	
	Missing	953 (1.5 %)	938 (1.6 %)	15 (0.9 %)	
Chest x-ray	Not done	9409 (15.2 %)	9075 (15.8 %)	334 (20.9 %)	<0.001
	Normal	2107 (3.4 %)	2049 (3.6 %)	58 (3.6 %)	
	Additional pathology	245 (0.4 %)	237 (0.4 %)	8 (0.5 %)	
	Suggestive of TB	35221 (57.0 %)	34295 (59.8 %)	926 (58.0 %)	
	Cavitation	11995 (19.4 %)	11725 (20.4 %)	270 (16.9 %)	
	Missing	2840 (4.6 %)	2710 (4.5 %)	130 (7.5 %)	
Microbiological status	Negative	8787 (15.0 %)	8647 (15.2 %)	140 (8.4 %)	<0.001
	Positive	49844 (85.0 %)	48318 (84.8 %)	1526 (91.6 %)	
	Missing	3186 (5.2 %)	3126 (5.2 %)	60 (3.5 %)	
Initial drug scheme	Other	1361 (2.2 %)	1232 (2.1 %)	129 (7.5 %)	<0.001
	RHZ	11356 (18.4 %)	11101 (18.5 %)	255 (14.7 %)	
	RHZE	49100 (79.4 %)	47758 (79.5 %)	1342 (77.8 %)	
Directly observed treatment-DOT	No	16219 (26.5 %)	15724 (26.4 %)	495 (30.4 %)	<0.001
	Yes	45050 (73.5 %)	43918 (73.6 %)	1132 (69.6 %)	
	Missing	548 (0.9 %)	449 (0.8 %)	99 (5.7 %)	

*PHC* primary health clinics, *R* rifampicin, *H* isoniazid, *Z* pyrazinamide, *E* ethambutol

Almost two thirds of patients were diagnosed outside hospitals. However, homeless individuals were frequently diagnosed at emergency services (34.7 %) (P < 0.001). In terms of chest x-rays at diagnosis, 76.4 % had positive findings for PTB. Microbiologic confirmation diagnosis occurred more frequently among the homeless (91.6 % vs. 84.8 %, P < 0.001). The majority of patients (approximately 80 %) were treated with the four drugs scheme (RHZE). DOT was offered to 73.5 % of patients overall and to 69.6 % of homeless patients (P < 0.001).

### Homelessness and treatment outcomes

The treatment outcomes are shown in Table 2. The percentage of treatment success among all cases was high: 81.4 % (95 % CI, 81.1-81.7). Among homeless patients the percentage of unsuccessful outcome of treatment was 57.3 % (OR 6.32, 95 % CI 5.73-6.97, P < 0.001). Loss to follow-up (39.0 %) and death (10.5 %) were the main problems.

**Table 2:** Treatment outcomes among 61,817 newly diagnosed PTB and their distribution among patients who were homeless and those who were not

Treatment outcomes^a^	Overall	Not Homelessness	Homelessness	P value
	(n = 61817)	(n = 60091)	(n = 1726)	
				<0.001
Treatment success	50302 (81.4 %)	49565 (82.5 %)	737 (42.7 %)	
Treatment failure	374 (0.6 %)	367 (0.6 %)	7 (0.4 %)	
Death	3800 (6.2 %)	3619 (6.0 %)	181 (10.5 %)	
Loss to follow-up	6307 (10.2 %)	5633 (9.4 %)	674 (39.0 %)	
Not evaluated	1034 (1.7 %)	907 (1.5 %)	127 (7.4 %)	

^a^Treatment outcome definitions are in the online material

After full adjustment, our model in the complete case analysis evaluated 36,604 (59 %) patients, as shown in Table 3. Homelessness remained strongly associated with poor outcomes (aOR 4.96, 95 % CI 4.27-5.76, P < 0.001). The influence of age in the unsuccessful results of treatment was non-linear, with older patients being associated with higher odds of poor outcomes (P < 0.001). Being male, non-white and non-Brazilian were associated with worse outcomes. Higher level of education was non-linearly associated with better outcomes, with strong protective impact after eight years of education. Alcohol and drug use had a marked effect on unsuccessful outcomes of treatment (aOR 1.33, 95 % CI 1.23-1.44, P < 0.001 and aOR 2.06, 95 % CI 1.89-2.24, P < 0.001, respectively). After adjustment, diabetes mellitus, mental disorders and other immunosuppression showed no evidence of association with unsuccessful outcomes of treatment. Patients diagnosed at emergency services or when hospitalized had worse outcomes (aOR 1.54, 95 % CI 1.44-1.65 and aOR 1.89, 95 % CI 1.73-2.06, P < 0.001, respectively). Analysis of chest x-ray pattern at diagnosis showed that patients with cavitation had better outcomes than patients who did not have a chest x-ray. Patients with confirmed microbiological diagnosis had an aOR of 0.92 (95 % CI 0.85-1.00, P = 0.048) for unsuccessful outcomes of treatment. Finally, patients who received the recommended drugs (triple until 2009 or quadruple after 2010) and those who received DOT had better outcomes. There was no evidence of multicollinearity in the final model.

**Table 3:** Fully adjusted estimates for the association between homelessness and unsuccessful outcome of treatment of newly diagnosed PTB by logistic regression model (n = 36,604, complete case analysis)

		Values	Adjusted OR (95 % CI)	P value
Exposure	Homelessness	No	Reference	<0.001
		Yes	4.96 (4.27-5.76)	
Adjusted for	Age, years	15-25	Reference	<0.001
		25.1-35	1.01 (0.93-1.10)	P_dep_ < 0.001
		35.1-45	0.93 (0.85-1.02)	
		45.1-55	0.84 (0.76-0.93)	
		55.1-65	0.94 (0.83-1.06)	
		65.1-75	1.08 (0.92-1.27)	
		75.1-85	1.92 (1.56-2.36)	
		85.1-105	2.47 (1.62-3.76)	
	Gender	Female	Reference	<0.001
		Male	1.32 (1.23-1.41)	
	Country of birth	Brazil	Reference	<0.001
		Not-Brazil	2.09 (1.74-2.51)	
	Race	White	Reference	0.001
		Black	1.16 (1.06-1.28)	
		Mixed/Brown	1.08 (1.01-1.15)	
		Asian	0.82 (0.59-1.13)	
		Indigenous	1.48 (1.10-1.99)	
	Education	Illiterate	Reference	<0.001
		1-3 years	1.04 (0.88-1.23)	P_dep_ < 0.001
		4-7 years	1.07 (0.92-1.25)	
		8-11 years	0.84 (0.72-0.99)	
		12-14 years	0.57 (0.46-0.70)	
		> = 15 years	0.62 (0.48-0.81)	
	Alcohol	No	Reference	<0.001
		Yes	1.33 (1.23-1.44)	
	Diabetes mellitus	No	Reference	0.173
		Yes	0.92 (0.81-1.04)	
	Drug users	No	Reference	<0.001
		Yes	2.06 (1.89-2.24)	
	Mental disorder	No	Reference	0.441
		Yes	1.10 (0.87-1.38)	
	Other immunosuppression	No	Reference	0.144
		Yes	1.29 (0.92-1.79)	
	Place of diagnosis	PHC/Ambulatory	Reference	<0.001
		Emergency service	1.54 (1.44-1.65)	
		Hospital	1.89 (1.73-2.06)	
	Chest x-ray	Not done	Reference	<0.001
		Normal	0.96 (0.80-1.15)	
		Additional pathology	1.07 (0.64-1.79)	
		Suggestive of TB	1.04 (0.95-1.14)	
		Cavitation	0.86 (0.78-0.96)	
	Microbiologic status	Negative	Reference	0.048
		Positive	0.92 (0.85-1.00)	
	Initial drug scheme	Other	Reference	<0.001
		RHZ	0.73 (0.59-0.91)	
		RHZE	0.65 (0.53-0.80)	
	Directly observed treatment-DOT	No	Reference	<0.001
		Yes	0.45 (0.42-0.48)	

*OR* odds ratio, *CI* confidence interval, *P*_*dep*_ test for departure from linearity, *PHC* primary health clinics, *R* rifampicin, *H* isoniazid, *Z* pyrazinamide and *E* ethambutol

### Sensitivity analysis in multiple imputed data

We observed missing values for eight variables: age (<0.1 %), DOT (0.9 %), place of diagnosis (1.5 %), chest x-ray (4.6 %), microbiologic status (5.2 %), race (15.5 %), country of birth (16.2 %) and education level (19.5 %). The multiple imputation process had good convergence as evaluated by the WLF.

Homelessness remained strongly associated with unsuccessful outcome of treatment (aOR 4.81, 95 % CI 4.33-5.35, P < 0.001) after full adjustment in multiple imputed analyses (Additional file 1: eTable 2). There were almost no changes in the point-estimate values for aORs compared with the complete case analysis. However, some covariates presented strong evidence of association not observed in the complete case analysis, such as diabetes mellitus, other immunosuppression and microbiologic diagnosis (Additional file 1: eTable 2).

### Interaction with HIV status

**Fig. 3:**
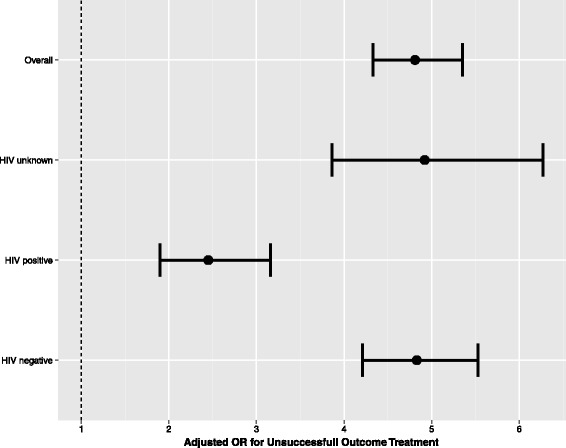
Effect modification by HIV status on the association between homelessness and the unsuccessful outcome of pulmonary TB treatment. Results from the fully adjusted model (adjusted for age, gender, country of birth, race, education level, alcohol and drug use, diabetes mellitus, mental disorder, immunosuppression other than HIV, place of diagnosis, chest x-ray and microbiologic status at diagnosis, initial drug scheme and directly observed-treatment-DOT) in the multiple imputed datasets. P value for interaction < 0.001 (Additional file 1: eTable 3)

For the evaluation of the interaction effect on the fully adjusted model, we used the multiple imputed datasets to increase power. HIV status had a marked effect modification for homelessness (P_interaction_ < 0.001 for interaction), changing its association among HIV positive patients (aOR 2.45, 95 % CI 1.90-3.16) (Fig. 3, Additional file 1: eTable 3).

### Population attributable fraction-PAF

From the final model of the complete case analysis, we estimated the PAF for homelessness. Because homelessness was strongly associated with alcohol and drug use (Table 4), we estimated a joint PAF for these three factors. When combined, their PAF totalled nearly 20.0 %.

**Table 4:** Joint population attributable fraction for impact of homelessness, alcohol and drug use on the unsuccessful outcome of treatment of newly diagnosed PTB cases (n = 61,817 cases)

Homelessness	Alcohol	Drug users	Number (%)	Odds ratio to unsuccessful outcome of treatment	Proportion among unsuccessful outcome of treatment -%	PAF for unsuccessful outcome of treatment -%
No	No	No	48171 (77.9 %)	Reference	65.0 %	
No	No	Yes	3279 (5.3 %)	2.33	7.7 %	4.4 %
No	Yes	No	6263 (10.1 %)	1.52	12.3 %	4.2 %
No	Yes	Yes	2378 (3.9 %)	2.56	6.4 %	3.9 %
Yes	No	No	788 (1.3 %)	6.25	3.9 %	3.3 %
Yes	No	Yes	192 (0.3 %)	16.7	1.2 %	1.1 %
Yes	Yes	No	417 (0.7 %)	5.56	1.8 %	1.5 %
Yes	Yes	Yes	329 (0.5 %)	9.09	1.7 %	1.5 %

*PTB* pulmonary tuberculosis, *PAF* population attributable fraction

## Discussion

Our study showed that 2.8 % (95 % CI 2.7-2.9 %) of newly diagnosed PTB patients in SPS during 2009-2013 were homeless. Homelessness had a marked association with unsuccessful outcome of treatment (aOR 4.96, 95 % CI 4.27-5.76, P < 0.001) after adjusting for several confounding factors; we found similar results in the multiple imputed data analysis. Losses to follow-up and deaths were the main poor outcomes for the homeless. Homelessness, alcohol use and drug use had a joint PAF of ~20 %. Interestingly, HIV status exerted an effect modification on the association between homelessness and unsuccessful outcome of treatment.

Unsuccessful outcome of treatment among homeless patients was very high (57.3 %) in our analysis, comparable to a cohort study in London in 2003 [26]. The association of homelessness with poor outcomes has been strong in developed countries [42, 49–52], achieving an aOR of 9.91 (95 % CI 4.38-22.38) in an Italian cohort with 3.8 % (60/1564) prevalence of homelessness [53]. In contrast, a Spanish cohort showed no association between homelessness (prevalence ~2.1 %) and poor outcomes after adjustment for predominating age, alcohol use, drug use, immigration and HIV status [54]. In terms of low- and middle-income countries, a study in Nicaragua showed that homelessness was associated with loss to follow-up (n = 502, aOR 3.00, 95 % CI 1.44-6.23) [55]. In a small cross-sectional study from Colombia which analysed deaths in TB patients, homelessness was present in 22 % (12/55) of cases, the majority being diagnosed at hospital, suggesting late diagnosis [56].

Two overlapped population-based cohorts from the USA (1994-2003 and 1994-2010), similar in approach to our study, reported a prevalence of homelessness around 6 % among TB cases [24, 25]. Homeless cases had characteristics comparable to our cohort: young adult males, high prevalence of alcohol and drug abuse and high prevalence of HIV positivity. In the 1994-2003 cohort, treatment success was 77 % for homelessness vs. 84 % for non-homelessness. DOT coverage was 86 % and homeless patients who received DOT were more likely to complete treatment [25]. In the 1994-2010 cohort, homelessness had twice the odds for unsuccessful outcome of treatment [24]. Both USA cohorts included PTB, EPTB and re-treatment cases. Although there were similarities with our population, homelessness was associated with unsuccessful outcome of treatment to a much lower degree in the USA. Improved social support [24, 25, 27, 57] and campaigns to improve housing during treatment [58] could explain these differences and show that the challenge of successful treatment of tuberculosis among the homeless can be met.

Our final model was generally consistent with the literature, highlighting the impact of important features on TB treatment outcomes. Diabetes mellitus was associated with better outcomes in the multiple imputed analysis, although in the literature it has been associated with death and treatment failure [20]. We believe that putting together all the “undesired outcomes” as unsuccessful outcome of treatment, we were unable to observe this association, as diabetes would decrease cure and increase mortality but not increase loss to follow-up. In fact, diabetes patients are likely to be followed up for medical treatment. The influence of microbiological confirmation [12, 15, 19] and chest x-ray [12] are controversial, with studies showing different associations for TB outcomes. Interestingly, we found that the effect of homelessness was less pronounced among HIV positive patients. We hypothesized that for these patients, PTB treatment was prioritized, leading to better management and follow-up by the TB and HIV-AIDS program than for HIV negative or unknown groups.

This study analysed a large dataset that covers all of SPS (41 million inhabitants). We followed our a priori plan of analysis, using multiple imputation to deal with missing data. We estimated the PAF for vulnerable conditions, a cornerstone of public health decisions. Few studies have addressed this issue, although PAF is fundamental to addressing the “social face” of TB [2, 3, 23]. Assuming a causal association between lower treatment success and homelessness, alcohol use and drug use, we estimated around 20 % of unsuccessful outcome of treatment in our population would be prevented if we could eliminate these exposures, or the increased risk associated with them.

The study has some limitations. First, the ascertainment of homelessness is unlikely to have false-positives. However, we cannot be confident that no patients pretended not to be homeless for reasons of stigmatisation. Therefore, we could have underestimated the prevalence and the impact of homelessness in our study. Second, we analysed only newly diagnosed PTB, which strengthened our internal validity but could have decreased our perception of the TB burden due to homelessness. Indeed, it is likely that homelessness prevalence is higher among relapses, re-treatments and other clinical forms of TB. Our aim was to provide data to plan interventions; therefore, we restricted our analysis to pulmonary cases, which are responsible for most transmission and the main burden of TB. Third, we applied the WHO definition for HIV status, which could be an issue because “HIV unknown” would include both positive and negative patients [17]. However, this was less important because of the high HIV testing in our population. The factors beyond not testing for HIV in PTB patients should be further explored. Fourth, by grouping the negative outcomes as unsuccessful outcome of treatment, we were not able to differentiate the effect on specific negative outcomes. The pragmatic approach of our analysis using routine data to inform stakeholders justifies our analysis; however, we believe that TB outcomes definitions and means of analysing them should be improved.

To improve TB health care for vulnerable groups is a difficult task which requires multifaceted interventions [22, 31, 59], involving governmental and community actions. There are several barriers, from finance to human support [59]. Indeed, adherence to TB treatment is strongly influenced by disease awareness and stigmatisation [30]. Additionally, TB treatment is associated with high direct/indirect costs [6], as social support is needed to achieve compliance [4]. Some interventions have had promising results for LTBI treatment in homeless patients, in addition to nurse case management with educational programs and incentives [60] and monetary incentives alone [61, 62]. For PTB treatment, focus on DOT strategy together with incentives at each visit and bonuses after completion were associated with positive results in the USA [8, 27]. In Japan, the combination of DOT with social support was associated not only with better outcomes but also empowerment of homeless patients [63].

Brazil has a national universal health coverage system and TB treatment is fully provided for free by the TB Program. Important improvements in the program have been made, although a special focus on the homeless is needed. We observed a high proportion of homeless patients being diagnosed at hospitals. Together with other important factors for poor outcomes, we propose that homeless patients are more likely to have delayed diagnosis and worse access to health care [64]. Active case finding in the homeless population and at shelter admission could be an effective strategy to tackle delayed diagnosis [65]. Furthermore, we observed that the proportion of homeless patients who received DOT was lower than non-homeless patients, although we had expected the contrary. The implementation of a specific campaign for DOT among the homeless, additionally providing socio-economic support might be a feasible and effective strategy to achieve better treatment outcomes in this vulnerable group [27, 57]. Structural and social transformations are necessary, improving not only TB treatment, but decreasing TB incidence in this population. Conditional cash transfer programs [66], food provision during treatment [40] and mobile health units for care of the homeless are promising in this setting [67].

Vulnerable conditions are so important for TB that guidelines and policy reports have launched specific documents for vulnerable groups [29, 30]. It is important to discuss the ethical issues surrounding TB, vulnerable groups and the implemented strategies [68].

## Conclusions

In our study we reported the main features of PTB among the homeless and found evidence that homelessness plays an important role in the PTB burden in a middle-income country such as Brazil. We believe that specific local policies dedicated to this vulnerable group and TB are fundamental and should be further discussed and implemented.

### Ethical consideration

We had official written permission from the Data Guardians, Health Department of SPS (21/05/2014) and Ethical Approval from the local Ethics Committee (03/09/2014, CAPPEsq-270/14) and from the LSHTM Ethics Committee (05/05/2015, Ref 9754).

## Additional file

Additional file 1:
**eTables 1.** Adapted from 2013 WHO Definitions and reporting framework for tuberculosis – 2013 revision (updated December 2014). **eTable 2.** Fully adjusted estimates for association between homelessness and the unsuccessful outcome of treatment of newly diagnosed PTB by logistic regression model in five multiple imputed datasets (n=61,817). **eTable 3.** Interaction of HIV status in the association between homelessness and the unsuccessful outcome of treatment of newly diagnosed PTB by fully-adjusted logistic regression model in 5 multiple imputed datasets (n=61,817). (DOCX 35 kb)

## Abbreviations

DOT: Directly observed-treatment
EPTB: Extra-pulmonary tuberculosis
HDI: Human development index
LTBI: Latent tuberculosis infection
MAR: Missing at random
OR: Odds ratio
PAF: Population attributable fraction
PTB: Pulmonary tuberculosis
RHZE: Rifampicin + isoniazid + pyrazinamide + ethambutol
SPS: São Paulo State
TB: Tuberculosis
WHO: World Health Organization
WLF: Worst linear function
