# Supplementary material for: The impact of being homeless on the unsuccessful outcome of treatment of pulmonary TB in São Paulo State, Brazil
Source: BMC Med. 2016 Mar 23;14:41. doi: 10.1186/s12916-016-0584-8 (PMC4804546; doi:10.1186/s12916-016-0584-8)
Supplement: Additional file 1: — eTables 1. Adapted from 2013 WHO Definitions and reporting framework for tuberculosis – 2013 revision (updated December 2014). eTable 2. Fully adjusted estimates for association between homelessness and the unsuccessful outcome of treatment of newly diagnosed PTB by logistic regression model in five multiple imputed datasets (n=61,817). eTable 3. Interaction of HIV status in the association between homelessness and the unsuccessful outcome of treatment of newly diagnosed PTB by fully-adjusted logistic regression model in 5 multiple imputed datasets (n=61,817). (DOCX 35 kb) [file 12916_2016_584_MOESM1_ESM.docx]

Online supplementary data

# The impact of being homeless on the unsuccessful outcome of treatment of pulmonary TB in São Paulo State, Brazil

Otavio T. Ranzani, Carlos R. R. Carvalho, Eliseu A. Waldman, Laura C. Rodrigues

eTables 1. **Adapted from 2013 WHO Definitions and reporting framework for tuberculosis – 2013 revision (updated December 2014)**

**Other definitions and Data Management**

eTable 2. **Fully adjusted estimates for association between homelessness and the unsuccessful outcome of treatment of newly diagnosed PTB by logistic regression model in five multiple imputed datasets (n=61,817)**

eTable 3. **Interaction of HIV status in the association between homelessness and the unsuccessful outcome of treatment of newly diagnosed PTB by fully-adjusted logistic regression model in 5 multiple imputed datasets (n=61,817)**

eTables 1. **Adapted** **2013 WHO Definitions and reporting framework for tuberculosis – 2013 revision (updated December 2014)[1]**

**Presumptive TB** refers to a patient who presents with symptoms or signs suggestive of TB (previously known as a TB suspect)

**Case definitions - A.1**

| **Case definition** | **Explanation** |
| --- | --- |
| Bacteriologically confirmed TB case | is one from whom a biological specimen is positive by smear microscopy, culture or WRD (such as Xpert MTB/RIF). All such cases should be notified, regardless of whether TB treatment has started |
| Clinically diagnosed TB case | is one who does not fulfil the criteria for bacteriological confirmation but has been diagnosed with active TB by a clinician or other medical practitioner who has decided to give the patient a full course of TB treatment. This definition includes cases diagnosed on the basis of X-ray abnormalities or suggestive histology and extrapulmonary cases without laboratory confirmation. Clinically diagnosed cases subsequently found to be bacteriologically positive (before or after starting treatment) should be reclassified as bacteriologically confirmed |

**Classification based on anatomical site of disease - A.1.1**

| Classification | Definition |
| --- | --- |
| Pulmonary tuberculosis (PTB) | refers to any bacteriologically confirmed or clinically diagnosed case of TB involving the lung parenchyma or the tracheobronchial tree. Miliary TB is classified as PTB because there are lesions in the lungs. Tuberculous intra-thoracic lymphadenopathy (mediastinal and/or hilar) or tuberculous pleural effusion, without radiographic abnormalities in the lungs, constitutes a case of extrapulmonary TB. A patient with both pulmonary and extrapulmonary TB should be classified as a case of PTB |
| Extrapulmonary tuberculosis (EPTB) | refers to any bacteriologically confirmed or clinically diagnosed case of TB involving organs other than the lungs, e.g. pleura, lymph nodes, abdomen, genitourinary tract, skin, joints and bones, meninges |

**Classification based on history of previous TB treatment – item A.1.2**

| Classification | Definition |
| --- | --- |
| New patients | have never been treated for TB or have taken anti-TB drugs for less than 1 month |
| Previously treated patients | have received 1 month or more of anti-TB drugs in the past |
| Relapse patients | have previously been treated for TB, were declared cured or treatment completed at the end of their most recent course of treatment, and are now diagnosed with a recurrent episode of TB (either a true relapse or a new episode of TB caused by reinfection) |
| Treatment after failure patients | are those who have previously been treated for TB and whose treatment failed at the end of their most recent course of treatment |
| Treatment after loss to follow-up patients | have previously been treated for TB and were declared lost to follow-up at the end of their most recent course of treatment. (These were previously known as treatment after default patients.) |
| Other previously treated patients | are those who have previously been treated for TB but whose outcome after their most recent course of treatment is unknown or undocumented |
| Patients with unknown previous TB treatment history do not fit into any of the categories listed above. New and relapse cases of TB are incident TB cases | |

**Classification based on HIV status – item A.1.3**

| **Classification** | **Definition** |
| --- | --- |
| HIV-positive TB patient | refers to any bacteriologically confirmed or clinically diagnosed case of TB who has a positive result from HIV testing conducted at the time of TB diagnosis or other documented evidence of enrolment in HIV care, such as enrolment in the pre-ART register or in the ART register once ART has been started |
| HIV-negative TB patient | refers to any bacteriologically confirmed or clinically diagnosed case of TB who has a negative result from HIV testing conducted at the time of TB diagnosis. Any HIV-negative TB patient subsequently found to be HIV-positive should be reclassified accordingly |
| HIV status unknown TB patient | refers to any bacteriologically confirmed or clinically diagnosed case of TB who has no result of HIV testing and no other documented evidence of enrolment in HIV care. If the patient’s HIV status is subsequently determined, he or she should be reclassified accordingly |

.

**Adapted TB Outcomes from WHO (not includes resistant TB) – item A.2.1**

| **Outcome** | **Definition** |
| --- | --- |
| Cured | A pulmonary TB patient with bacteriologically confirmed TB at the beginning of treatment who was smear- or culture-negative in the last month of treatment and on at least one previous occasion |
| Completed treatment | A TB patient who completed treatment without evidence of failure but with no record to show that sputum smear or culture results in the last month of treatment and on at least one previous occasion were negative, either because tests were not done or because results are unavailable |
| Treatment failed | For the São Paulo State TB-Program, treatment failure was defined as any proven resistance to TB drugs during the treatment. |
| Died | A TB patient who dies for any reason before starting or during the course of treatment |
| Lost to follow-up | A TB patient who did not start treatment or whose treatment was interrupted for 2 consecutive months or more |
| Not evaluated | A TB patient for whom no treatment outcome is assigned. This includes cases “transferred out” to another treatment unit as well as cases for whom the treatment outcome is unknown to the reporting unit |
| Treatment success | The sum of cured and treatment completed |
| Cohort | A group of patients in whom TB has been diagnosed.  This group forms the denominator for calculating treatment outcomes. The sum of the above treatment outcomes, plus any cases for whom no outcome is recorded (including those ‘still on treatment’) and “transferred out” cases should equal the number of cases registered. Some countries monitor outcomes among cohort defined by sputum smear and/or culture, and define cure and failure according to the best laboratory evidence available for each patient. |

**Other definitions:**

Variable: **Other immunosuppression**

We pre-specified that the variable “Other immunosuppression” could also be a potential effect modifier, but with much weaker effect. Therefore, we choose not to group those with HIV positivity, and, due to small number of patients in this category, we did not explore this potential interaction in this study.

**Data management**

TBweb was constructed with a user-friendly interface for data entry, with drop-down lists and hierarchical variables. The database has also a unique identifier for each treatment, avoiding duplications during the same treatment (a common problem for transferences from one health unit to another). The raw database was exported after Ethical approval and it was extensively checked for consistency, implausible values and typos.

eTable 2. Fully adjusted estimates for the association of homelessness with the unsuccessful outcome of treatment of newly diagnosed PTB by logistic regression model in five multiple imputed datasets (n=61,817)

|  | **Variable** |  | **Values** | **Adjusted**  **OR (95% CI)** | **P value** |
| --- | --- | --- | --- | --- | --- |
| **Exposure** | **Homelessness** | | No | Reference |  |
|  |  |  | Yes | 4.81 (4.33-5.35) | <0.001 |
| **Adjusted for** | Age, years | | 15-25 | Reference |  |
|  |  | | 25.1-35 | 1.08 (1.01-1.15) | 0.027 |
|  |  | | 35.1-45 | 1.02 (0.95-1.10) | 0.515 |
|  |  | | 45.1-55 | 0.97 (0.90-1.05) | 0.510 |
|  |  | | 55.1-65 | 1.05 (0.96-1.14) | 0.332 |
|  |  | | 65.1-75 | 1.36 (1.21-1.52) | <0.001 |
|  |  | | 75.1-85 | 2.26 (1.96-2.61) | <0.001 |
|  |  | | 85.1-105 | 3.67 (2.77-4.87) | <0.001 |
|  | Gender | | Female | Reference |  |
|  |  | | Male | 1.29 (1.23-1.36) | <0.001 |
|  | Country of birth | | Brazil | Reference |  |
|  |  | | Not-Brazil | 2.02 (1.74-2.34) | <0.001 |
|  | Race | | White | Reference |  |
|  |  | | Black | 1.20 (1.10-1.31) | <0.001 |
|  |  | | Mixed/Brown | 1.11 (1.04-1.18) | 0.003 |
|  |  | | Asian | 0.79 (0.62-1.01) | 0.055 |
|  |  | | Indigenous | 1.42 (1.10-1.83) | 0.008 |
|  | Education | | Illiterate | Reference |  |
|  |  | | 1-3 years | 1.03 (0.88-1.21) | 0.668 |
|  |  | | 4-7 years | 1.07 (0.94-1.23) | 0.320 |
|  |  | | 8-11 years | 0.86 (0.75-0.98) | 0.028 |
|  |  | | 12-14 years | 0.58 (0.49-0.68) | <0.001 |
|  |  | | >= 15 years | 0.57 (0.46-0.70) | <0.001 |
|  | Alcohol | | No | Reference |  |
|  |  | | Yes | 1.31 (1.24-1.39) | <0.001 |
|  | Diabetes mellitus | | No | Reference |  |
|  |  | | Yes | 0.86 (0.79-0.95) | 0.002 |
|  | Drug users | | No | Reference |  |
|  |  | | Yes | 2.00 (1.86-2.13) | <0.001 |
|  | Mental disorder | | No | Reference |  |
|  |  | | Yes | 1.14 (0.98-1.32) | 0.098 |
|  | Other immunosuppression | | No | Reference |  |
|  |  | | Yes | 1.62 (1.30-2.02) | <0.001 |
|  | Place of diagnosis | | PHC/Ambulatory | Reference |  |
|  |  | | Emergency service | 1.62 (1.30-2.02) | <0.001 |
|  |  | | Hospital | 2.06 (1.94-2.19) | <0.001 |
|  | Chest-X-ray | | Not done | Reference |  |
|  |  | | Normal | 1.09 (0.95-1.24) | 0.231 |
|  |  | | Additional pathology | 1.37 (1.01-1.85) | 0.042 |
|  |  | | Suggestive of TB | 1.04 (0.97-1.11) | 0.286 |
|  |  | | Cavitation | 0.86 (0.79-0.95) | <0.001 |
|  | Microbiologic status | | Negative | Reference |  |
|  |  | | Positive | 0.89 (0.84-0.95) | <0.001 |
|  | Initial drug scheme | | Other | Reference |  |
|  |  | | RHZ | 0.45 (0.40-0.51) | <0.001 |
|  |  | | RHZE | 0.40 (0.35-0.45) | <0.001 |
|  | Directly observed treatment-DOT | | No | Reference |  |
|  |  | | Yes | 0.43 (0.41-0.45) | <0.001 |

OR-odds ratio, CI- confidence interval, P_dep_-test for departure from linearity, PHC-primary health clinics, R-Rifampicin, H-Isoniazid, Z-Pyrazinamide and E-Ethambutol

eTable 3. **Interaction of HIV status in the association between the exposures homelessness and the unsuccessful outcome of treatment of newly diagnosed PTB by fully-adjusted logistic regression model in 5 multiple imputed datasets (n=61,817)**

| **Stratified by HIV status** | **Exposure** | **S-S adjusted* OR for treatment success** | **P-value for interaction** |
| --- | --- | --- | --- |
|  | **Homeless** |  | **<0.001** |
| Positive | Not-Homelessness | Reference |  |
|  | Homelessness | 2.45 (1.90-3.16) |  |
| Negative | Not-Homelessness | Reference |  |
|  | Homelessness | 4.83 (4.21-5.53) |  |
| Unknown | Not-Homelessness | Reference |  |
|  | Homelessness | 4.92 (3.86-6.27) |  |

* Adjusted for age, gender, race, education, alcohol, diabetes, drug users, mental disorder, other immunosuppression, place of diagnosis, chest-X-ray, microbiologic status, initial drug scheme and DOT.

S-S adjusted OR-stratum-specific adjusted odds ratio, CI confidence interval, DOT-Directly observed treatment

References:

1. World Health Organization-WHO: **Definitions and reporting framework for tuberculosis - 2013 revision (updated December 2014)**. *WHO Publications* 2013:1-47.
